# Clustering by fast search and merge of local density peaks for gene expression microarray data

**DOI:** 10.1038/srep45602

**Published:** 2017-04-19

**Authors:** Rashid Mehmood, Saeed El-Ashram, Rongfang Bie, Hussain Dawood, Anton Kos

**Affiliations:** 1 College of Information Science and Technology, Beijing Normal University, Beijing, 100875, China; 2 Department of Computer Science and Information Technology, University of Management Sciences and Information Technology, Kotli Azad Kashmir, 11100, Pakistan; 3 National Animal Protozoa Laboratory and College of Veterinary Medicine, Agricultural University, Beijing 100193, China; 4 Faculty of Science, Kafr El-Sheikh University, Kafr El-Sheikh, Egypt; 5 Faculty of Computing and Information Technology, University of Jeddah, Jeddah, Saudi Arabia; 6 Faculty of Electrical Engineering, University of Ljubljana, Ljubljana, Slovenia

## Abstract

Clustering is an unsupervised approach to classify elements based on their similarity, and it is used to find the intrinsic patterns of data. There are enormous applications of clustering in bioinformatics, pattern recognition, and astronomy. This paper presents a clustering approach based on the idea that density wise single or multiple connected regions make a cluster, in which density maxima point represents the center of the corresponding density region. More precisely, our approach firstly finds the local density regions and subsequently merges the density connected regions to form the meaningful clusters. This idea empowers the clustering procedure, in which outliers are automatically detected, higher dense regions are intuitively determined and merged to form clusters of arbitrary shape, and clusters are identified regardless the dimensionality of space in which they are embedded. Extensive experiments are performed on several complex data sets to analyze and compare our approach with the state-of-the-art clustering methods. In addition, we benchmarked the algorithm on gene expression microarray data sets for cancer subtyping; to distinguish normal tissues from tumor; and to classify multiple tissue data sets.

Clustering algorithms aim to organize elements into disjoint groups on the basis of their resemblance. Several computational strategies has been proposed for clustering, however, each strategy has its own definition of cluster. Thus, the output of diverse clustering algorithms may differ even on the same data set. K-means^1^ and K-medoids^2^ characterize clusters by grouping elements, having minimum distance from their cluster center. The minimization of the within-cluster sum of squares is the objective function, which is iteratively optimized to obtain the effective candidates for cluster centers. The cluster members are assigned to cluster centers based on their minimum distance, therefore, these approaches could not find the arbitrary shaped clusters. Unlike, K-means and K-mediod, density based approaches can easily detect the arbitrary shaped clusters. In density-based spatial clustering of applications with noise (DBSCAN)^3^, with optimal parametric settings of algorithm, density maxima connected regions are merged into single cluster and noise is detected as points having low density than threshold value. However, the optimal parametric settings can be non-trivial^4^; a drawback does not exist in the mean-shift clustering^4^,^5^. In mean shift clustering, a similar group of points converging to a maximum density distribution make a cluster by consuming high computational cost. In clustering by fast search and find of density peaks (CDP)^4^, cluster centers are characterized as points with higher local density and having large distance from any other local density. CDP uses a decision graph based approach to identify cluster centers, in a more intuitive way as compared with K-means or K-mediods based approaches. After successful identification of cluster centers, assignation of points to cluster centers is made based on the nearest neighbor with higher density. Therefore, for more complex data sets, CDP behaves like K-means and K-mediods. Furthermore, in CDP, no generalized density estimation method is provided, and decision graph could not express the effective cluster centers in the presence of multiple density peaks within a cluster.

## Methods

In this section, the proposed clustering approach and the detailed description of used synthetic and real world data sets are listed to support the presented study.

Here, a new clustering approach is presented in this paper. Similar to K-mediods and CDP, it is based on distance between data points, and like DBSCAN, it also has the characteristic of density-connectivity. Unlike CDP, our approach detects all density regions to form local clusters, subsequently finds density connected local clusters, and then automatically merges the local clusters to form the arbitrary shaped clusters.

The presented algorithm is based on the assumptions that single or multiple local density function(s) constitute a cluster, where each local density maxima is surrounding by neighbors with lower density, and they are comparatively at large distance from any other point with a higher local density. In order to identify local clusters, for each data point *i*, we compute local density *ρ*_*i*_ and distance *δ*_*i*_ from point with higher density. Both quantities are calculated based on the distance between points. For each data point *i*, the local density is defined as follows:


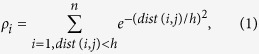


where, *h* is radius of the point *i*, which can be obtained using the heuristic approach given in ref. 4 to measure the cutoff distance, and *dist(i, j*) denotes the distance between points *i* and *j*. In Eq. 1, neighboring points within radius *h* are only considered to estimate the density of point *i*. Unlike CDP, where different methods are suggested to estimate the density, the effectiveness of presented approach does not rely on the nature of data set. Our clustering approach is only sensitive to relative magnitude of density, therefore, optimal results can be achieved with the appropriate choice of *h*. However, to measure the minimum distance *δ*_*i*_, between point *i* and the nearest point with higher density, we use similar approach like CDP, presented as follows:


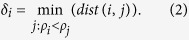


For points with the highest local density, *δ* will be much larger as compared to typical nearest neighbors. In next step, we employe *δ*_*i*_ and *ρ*_*i*_ to identify all of the local highest dense centers. Because of the unique characteristics, local cluster centers (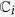) can be distinguish from ordinary cluster points using the Eq. 3, presented as follows:


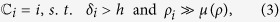


where, *μ(ρ*) is mean of *ρ*. After identification of 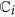, the rest of points are assigned to it in single step, based on the nearest neighbor of higher density concept^4^ to form the local clusters (*ψC*_*i*_). However, to overcome the risk of misclassification, a point *j* is directly reassigned to the nearest local cluster center if 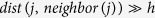. In order to merge *ψC*_*i*_, we identify the shared density region in *ψC*_*i*_, denoted as *λρ*_*i*_ and it contains all those points that are part of one cluster but also within *h* distance from some points belong to other clusters. Furthermore, for each *λρ*_*i*_, we discover the density maxima 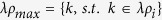, and merge two local clusters, if *λρ*_*max*_ satisfies the Eq. 4 that is presented as follows:


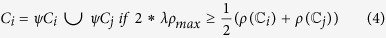


At last, we declare a point *x* as noise, if *ρ*_*x*_ is relatively very small as compared with average density of its belonging cluster and it is very far away from the rest of boundary points having average density. A comparison of time complexity of proposed method with famous clustering methods has presented in Table S11. Source code is available at http://bigdata.bnu.edu.cn/zh/clustering-1/.

### Data Sets

We selected 11 real-world data sets, comprises of 10 biomedical data sets (Table 1) and 1 human face detection data set, all cancer gene expression data sets are obtained from (http://portals.broadinstitute.org/cgi-bin/cancer/datasets.cgi). In, addition, 7 synthetic point distributions with different structures, complexities, and size are used.

## Results and Discussion

**Figure 1:**
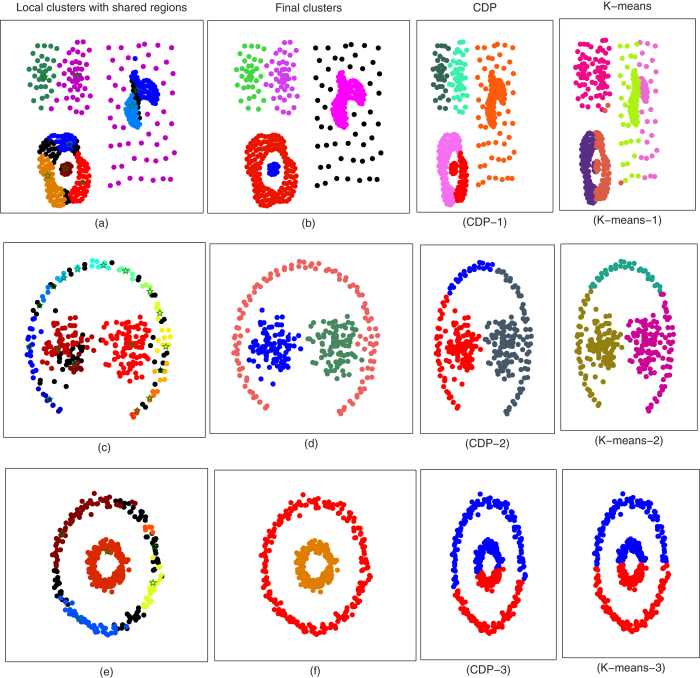
Clustering analysis and comparison of synthetic data sets. (**a**) Visualizes the performance of our algorithm to detect distinct densities in compound^6^ data set, star markers with different colors are referring to detected local clusters and shared density regions are visualized with black color. (**b**) After merging the local clusters, finalized clusters of (**a**) are shown. Even after optimization of input settings of CDP and K-means, assignation of points is not according to the visual intuition, as shown in (CDP-1, K-means-1). (**c**) 17 detected local clusters of path-based data set are visualized with star marker and black points are depicting shared density regions. (**d**) The proposed approach successfully merged the 17 local clusters into three clusters. (CDP-2, K-means-2) Three clusters by employing each comparative method are visualized, respectively. (**e**,**f**) The local and finalized clusters of toys problem data set are visualized. (CDP-3, K-means-3) CDP and K-means organized clusters of toys data sets are visualized, respectively.

**Figure 2:**
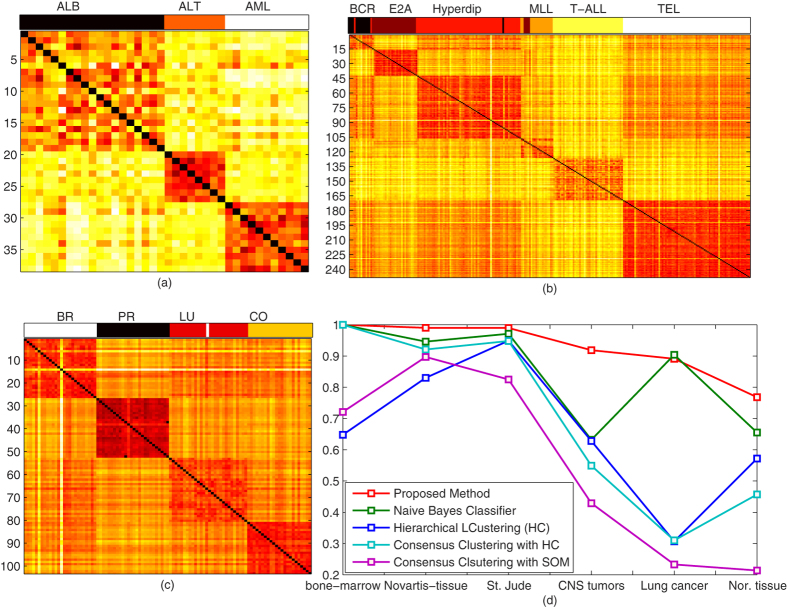
Clustering results for identification and organization of cancer subtypes from gene expression microarray databases. (**a**) Co-occurrence matrix of obtained subclasses of acute leukemia, where heatmap visualizes the inner class similarity. We organized 999 genes for 38 samples into 11 AML, 8 ALL, and 19 B-lineage sub classes with 100% accuracy. Color bar at the top of the image represents the class label of each element. (**b**) In St. Jude Leukemia data base, we successfully identified 6 prognostically important leukemia subtypes: T-lineage; ALL; E2A-PBX1; BCR-ABL; TEL-AML2; and MLL. (**c**) 103 samples of 1000 genes are clustered into 26 breast, 26 prostate, 28 lung, and 23 colon. Heatmap visualizes four distinct tissues classes in the co-occurrence matrix, and color bar represents the assigned class labels. (**d**) A Rand index based comparison of our method with nave-Bayes(NB) classifier, Hierarchical clustering (HC), consensus clustering with hierarchical clustering (CC_HC), and consensus clustering with (CC_SOM) is presented.

To explain the working of presented approach, first consider the test case as depicted in Fig. 1, where the approach is explained in two steps, and a visual comparison is made with K-means and CDP. In step 1, different colors present the locally detected clusters of compound data set^6^, where star markers depict the local cluster centers while shared density regions are marked with black color, as shown in Fig. 1(a). After merging the local detected clusters, six clusters are identified; including one noise cluster without having any cluster center. According to the survey^7^, F1 score of famous clustering algorithms such as CDP, DBSCAN, Hierarchical Clustering, and Spectral clustering for compound data set is less than 0.89. The optimal clusters, obtained from CDP and K-means are also visualized in Fig. 1 (CDP-1, K-means-1). Moreover, we evaluate the performance of the presented algorithm on another complex data set^8^. In Fig. 1(c), the locally detected clusters are visualized with different colors and black color is used to highlight the shared density regions. After merging procedure, three clusters are obtained with 100% accuracy, as shown in Fig. 2(d). However, at optimal settings, the output clusters by CDP and K-means are not compliant with visual intuition, as shown in Fig. 1 (CDP-2, K-means-2). To benchmark the proposed algorithm on nested structures, we tested the algorithm on toys data set obtained from ref. 9. Initially, we detected local clusters with overlapping regions, as illustrated in Fig. 1(e) and then regions are merged based on presented strategy, consequently, effective clusters are obtained as presented in Fig. 1(f). However, the CDP and K-means are less sensitive to detect overlapping structure from complex data sets, as visualized in Fig. 1 (CDP-3, K-means-3). In all these three test cases, a similar behavior of CDP and K-means is observed.

Next, the proposed approach is applied to other synthetic test cases, as presented in Fig. S1. In Fig. S1a, the evaluated results are comparable with the original method^4^. According to the ref. 7, the commonly used methods fail to organize FLAME^7^ into two clusters. Figure S1b presents the point distribution of A.K. jain’s toys problem^10^. A.K. Jain’s toys problem data set is actually comprised of two clusters, where regions with different densities exist, thus making it difficult for CDP and many famous clustering methods to successfully organize points into clusters. Initially, our algorithm detected 8 local clusters and subsequently merged them into two clusters, accurately. In Fig. S1c, we applied our approach on data set^4^, which was introduced to demonstrate the performance of spectral crusting^4^. Our approach accurately organized the data set into three distinct clusters. Furthermore, the evaluated results of aggregation data set^4^ are visualized in Fig. S1d.

We also tested the algorithm to the Olivetti Face Database^11^, a well-known benchmark in the field of machine learning algorithms, with interest to identifying number of subjects in the database, without any pre-training. This database has 40 ideal clusters and each cluster consists of 10 elements, thus, pose a serious challenge to identify the ideal distinct subjects, as the estimation of reliable densities is a difficult task^4^. At *h* = 1, our approach identified 64 numbers of distinct subjects, the pictorial clustering representation of first 120 images of database are shown in Fig. S2, where images with similar color belong to one cluster, gray images are the elements that are not assigned to any cluster, and blur gray images are misclassified. We classify 120 elements into 12 clusters with the accuracy of 85.8333%. However, CDP^4^ could organize only 42 images into 9 distinct subjects out of 100 first images of database, and rest of images remained unassign to any cluster. For all 400 images, CDP decision graph based approach could not allow to clearly recognize the number of clusters^4^. Unlike CDP, our approach automatically identified 64 clusters for full database with maximum accuracy.

To benchmark the proposed algorithm for the detection of the cancer subtypes, the separation of the normal lungs from tumors and the organization of the multi tissues into distinct classes, we utilized 10 genes expression databases, the detailed description of genes expressions databases is provided in Table 1.

For cancer subtyping, firstly, we applied the approach on leukemia gene expression microarray data set, to identify 11 acute myeloid leukemia (AML), 8 T-lineage acute lymphoblastic leukemia (ALL), and 19 B-lineage ALL samples. In order to achieve high accuracy, we normalized the data with Z-scoring^12^, and used cosine distance matrix to obtain the pairwise distances. The proposed algorithm detected three clusters with 100% accuracy, the obtained results are comparable to that of nave-Bayes (NB) classifier and Consensus clustering with HC^7^. A co-occurrence matrix of existing classes in data set is visualized using the heatmap, whereas, three obtained clusters are shown in form of color bar at top of the figure. The presented algorithm was also applied on St. Jude Leukemia data set to identify 6 prognostically important leukemia subtypes: T-lineage; ALL; E2A-PBX1; BCR-ABL; TEL-AML2; MLL. With 98.3871% accuracy, the proposed algorithm organized data set into six distinct leukemia sub classes. The obtained results are also comparable with that of NB even the classifier was trained on the same data set, the obtained subtypes of leukemia and the co-occurrence matrix of real classes are visualized in Fig. 2(b). To detect the distinct cancers (breast, prostate, lung, colon) from gene expression microarray, we also tested our algorithm on Novartis multi-tissue data set. It identified four distinct classes with accuracy of 99%, still higher accuracy as compared with NB and methods given in ref. 13. In Fig. 2(e), the superiority over the NB, HC, CC_*HC*_, and CC_*SOM*_ of our approach is illustrated. We used Rand Index (RI) measures to compare our approach with famous clustering methods used in ref. 13 for gene expression microarray. From Fig. 2(d), it can be observed that our method is more reliable to organize the genes into distinct subclasses. Our approach outperformed nave-Bayes classification, in which pre-training is also required for new data sets.

To benchmark the algorithm on high dimensional data sets, we tested it on gene expression data set of Normal Progenitor and Leukemic stem cell, obtained from ref. 14. This data set was classified into 5 distinct clusters and arranged cell types with 100% accuracy, as presented in Fig. S3. We also evaluated our method on CNS tumor gene expression data set, which posed a serious challenge to the famous clustering approaches^13^, the estimation of exact clusters and the points assignation was strenuous task^13^ even provided with small number of sample size for given classes. We successfully identified medulloblastomas (MD), malignant gliomas (Glio), atypical teratoid/rhabdoid tumors(Rhab), normal cerebellum (Ncer), and primitive neuroectodermal tumors (PNET), and classified with 0.9059 RI, however, RI for NB, HC, CC_HC^13^, CC_SOM^13^ is 0.655, 0.472, 0.572, and 0.487, respectively. In Fig. S4, distinct classes are visualized using the color bar, and a co-occurrence matrix of original clusters are also illustrated using the heatmap.

**Figure 3:**
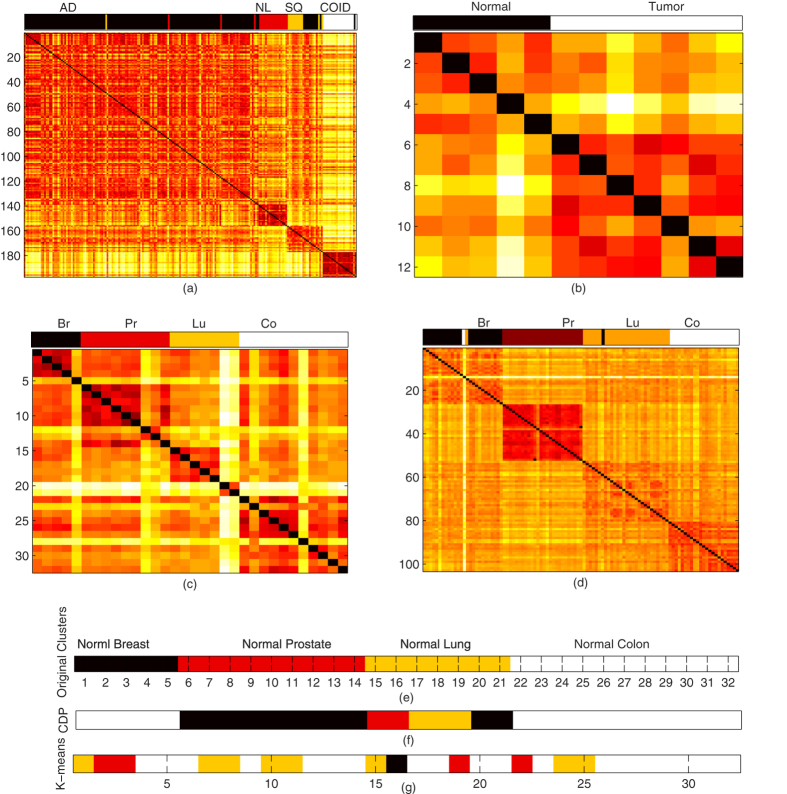
Clustering results for separating normal tissues from tumor, and classification of multi tissue, 1-channel microarray. (**a**) Lung cancer data set is organized into four distinct classes, with the accuracy of 98.4772%, normal lung is separated from tumor lung, however, we observe a high similarity between adenocarcinomas and squamous cell carcinomas as visualized in co-occurrence matrix, and color bar at top of co-occurrence matrix demonstrates the discovered classes. (**b**) The normal and tumor lung of mouse are visualized, with 100% accuracy, we organize the samples into normal and tumor lung. (**c**) Our approach, with accuracy of 97.1145%, find the four distinct tissue classes in the multi-a gene expression microarray. In multi-b tissue data set, we organize the whole data set into, 9 prostate (pr), 5 breast (br), 11 colon (co), and 7 lung (lu) normal tissues, accurately. (**e**) With different color schemes, distinct classes of multi-b are visualized, however, in (**f**,**g**) clusters of CDP and K-means are visualized at optimal parametric setting, respectively.

Next, we tested our algorithm on human and mouse lung gene expressions to detect and differentiate the normal lungs from tumor lungs. Firstly, we applied the algorithm on human lung cancer data set, obtained from ref. 15, and identified adenocarcinomas, squamous cell carcinomas, arcinoids, and normal lung tissues by using the 1000 genes for 197 samples. We achieved RI 0.8912 to organize the genes into five clusters, further, with accuracy of 98.4772%, we successfully differentiated normal tissue from tumor tissues, as shown in Fig. 3(a). In case of mouse samples obtained from ref. 16, there were only tumor lungs and normal lungs. With 100% accuracy, we found 5 normal lungs and 7 tumors, as shown in Fig. 3(b).

We also benchmark the presented approach on the multiple normal tissue types, high dimensional gene-expression 1-channel microarray data sets, obtained from two different generations of the Affymetrix Gene Chip oligonucleotide microarray platform, multi-a^17^ and multi-b^17^. Both data sets comprise of lung, prostate, colon, and breast types of tissues. In multi-a, our approach discovered distinct four categories of tissues and organized into 5 breast, 9 prostate, 7 lung, and 11 colon tissues, with 100% accuracy. In case of multi-a, we organized tissues into four categories, with accuracy of 98.0584%. For comparison, CDP and K-means clustering results of multi-a are visualized in Fig. 3(e,f), where each color represents a distinct class. At last, we also tested our approach to normal tissue data set, obtained from ref. 13, with the accuracy of 82.222%, we classified the data set into 13 distinct groups.

To cluster the gene expression microarray, mostly, we got the desirable results with normalization of Z-score, the normalization process and optimal values of *h* for each data set are provided in supplementary material, as Tables S1–S10. For gene expression data sets, the comparison of classification accuracy of the proposed method with state-of-the-art clustering methods has given in Table S12.

## Additional Information

**How to cite this article:** Mehmood, R. *et al*. Clustering by fast search and merge of local density peaks for gene expression microarray data. *Sci. Rep.* **7**, 45602; doi: 10.1038/srep45602 (2017).

**Publisher's note:** Springer Nature remains neutral with regard to jurisdictional claims in published maps and institutional affiliations.

## Supplementary Material

Supplementary Information

## Figures and Tables

**Table 1:** The detail description of genes expression data sets.

Data set	Objects	Features	Classes	References
Leukemia	38	999	3	7
Novartis multi-tissue	103	1000	4	13
St. Jude leukemia	248	985	6	13
CNS tumors	48	1000	5	18
Lung cancer	197	1000	4+	15
Multi-a	103	5565	4	17
Multi-b	34	5565	4	17
Normal tissues	99	1277	13	13
Normal progenitor and leukemic samples	22	22690	5	14
Mouse lung	12	217	4	16
