# Supplementary material for: Clustering by fast search and merge of local density peaks for gene expression microarray data
Source: Sci Rep. 2017 Apr 19;7:45602. doi: 10.1038/srep45602 (PMC5395818; doi:10.1038/srep45602)
Supplement: Supplementary Information [file srep45602-s1.doc]

**Clustering by fast search and merge of local density peaks for gene expression microarray data**

Rashid Mehmood^1,2,*^, Saeed El-Ashram^3,4^, Rongfang Bie^1^, Hussain Dawood^5^ & Anton Kos^6^

^1^College of Information Science and Technology, Beijing Normal University, Beijing, 100875, China

^2^Department of Computer Science and Information Technology, University of Management Sciences and Information Technology, Kotli Azad Kashmir, 11100, Pakistan

^3^National Animal Protozoa Laboratory and College of Veterinary Medicine, Agricultural University, Beijing 100193, China

^4^Faculty of Science, Kafr El-Sheikh University, Kafr El-Sheikh, Egypt

^5^Faculty of Computing and Information Technology, University of Jeddah, Jeddah, Saudi Arabia

^6^Faculty of Electrical Engineering, University of Ljubljana, Ljubljana, Slovenia.

[*corresponding.gulkhan007@gmail.com](mailto:*corresponding.gulkhan007@gmail.com)

**Supplementary Material**

Fig.S1: Point distribution from well-known synthetic structures. (a) We organized flame data set into two clustering at
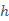
= 5.5. (b) In A.k Jain data set, there are more than one density maximum points that makes difficult for CDP to select appropriate cluster centers correctly, however, we successfully organized into two clusters at
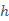
=8. (c) At
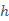
=4, we successfully clustered spiral data set into three clusters. (d) In case of aggregation data set, at
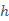
=3, we firstly identified 11 local clusters and then merged into seven finalized clusters.

Fig.S2: Pictorial presentation of 120 first images of Olivetti database^1^. Similar color images are belongs to same cluster, whereas gray images could not group into any clusters, and blur gray images are misclassified.

Fig.S3: In CNC tumors data set, algorithms, including HC, SOM, and K-means have very poor performance to find and cluster the data set into five distinct subclasses of tumor; however, our proposed method successfully identified five different subclasses with maximum accuracy. (a) The five different subtypes are graphically represented (heatmap) using the co-occurrence matrix of CNC tumors data set. (b) Different color codes are used to represent the identified five distinct subtypes of CNC tumors.

Fig.S4: with 100% accuracy, we identified and differentiate the Normal Progenitor and Leukemic samples.

**Table S1: Clustering Results of Leukemia (Bone Marrow)**

| Field Description |  |
| --- | --- |
| Sample | Human Leukemia |
| ALB ALT AML | Classification |
| Preprocessing and 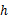 value | Normalize data with zscore and use 'cosine' distance at 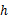= 9.4 |
| Accuracy | 100% |
| Sequence | 19 ALB,8 ALT, and AML |

**Table S2: Clustering Results of St. Jude Leukemia**

| Field Description |  |
| --- | --- |
| Sample | Human Leukemia |
| BCR E2A Hyperdip MLL T-ALL TEL | Classification |
| Preprocessing and 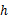 value | Normalize data with zscore and use 'Euclidean' distance at 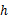 = 3.7 |
| Accuracy | 97.1774 % |
| Clusters Sequence  6 =BCR  1= E2A  2= Hyperdip  3= MLL  4= T-ALL  5= TEL | 6 6 6 6 **3** 6 6 6 6 6 6 6 6 6 6 **3** 1 1 1 1 1 1 1 1 1 1 1 1 1 1 1 1 1 1 1 1 1 1 1 1 1 1 2 2 2 2 2 2 2 2 2 2 2 2 2 2 2 2 2 2 2 2 2 2 2 2 2 2 2 2 2 2 2 2 2 2 2 2 2 2 2 2 2 2 2 2 2 2 2 2 2 2 2 2 2 **6** 2 2 2 2 2 2 2 2 2 2 3 3 **1 1 1 1** 3 3 3 3 3 3 3 3 3 3 3 3 3 3 4 4 4 4 4 4 4 4 4 4 4 4 4 4 4 4 4 4 4 4 4 4 4 4 4 4 4 4 4 4 4 4 4 4 4 4 4 4 4 4 4 4 4 5 5 5 5 5 5 5 5 5 5 5 5 5 5 5 5 5 5 5 5 5 5 5 5 5 5 5 5 5 5 5 5 5 5 5 5 5 5 5 5 5 5 5 5 5 5 5 5 5 5 5 5 5 5 5 5 5 5 5 5 5 5 5 5 5 5 5 5 5 5 5 5 5 5 5 5 5 5 5 |

**Table S3: Clustering Results of Novartis multi-tissue**

| Field Description |  |
| --- | --- |
| Sample | Novartis multi-tissue |
| BR PR LU CO | Classification |
| Preprocessing and 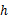 value | Normalize data with zscore and use 'Euclidean' distance at 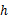 = 10 |
| Accuracy | 99.0291 % |
| Clusters Sequence  4= BR  1= PR  2= LU  3= CO | 4 4 4 4 4 4 4 4 4 4 4 4 4 4 4 4 4 4 4 4 4 4 4 4 4 4 1 1 1 1 1 1 1 1 1 1 1 1 1 1 1 1 1 1 1 1 1 1 1 1 1 1 2 2 2 2 2 2 2 2 2 2 2 2 2 **4** 2 2 2 2 2 2 2 2 2 2 2 2 2 2 3 3 3 3 3 3 3 3 3 3 3 3 3 3 3 3 3 3 3 3 3 3 3 |
| Note (the red color represents the misclassification sample) | |

**Table S4: Clustering Results of Normal Progenitor And Leukemic samples**

| Field Description |  |
| --- | --- |
| Sample | Normal Progenitor And Leukemic |
|  |  |
| Preprocessing and 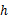 value | Use 'Euclidian' distance at 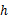 = 6.8 |

| Sample | **Clusters** | **Corrected?** |
| --- | --- | --- |
| HSC-2 | 1 | Yes |
| HSC-3 | 1 | Yes |
| HSC-4 | 1 | Yes |
| HSC-6 | 1 | Yes |
| HSC-5 | 1 | Yes |
| **CMP-2** | 2 | Yes |
| **CMP-3** | 2 | Yes |
| **CMP-4** | 2 | Yes |
| **CMP-1** | 2 | Yes |
| **GMP-2** | 3 | Yes |
| **GMP-3** | 3 | Yes |
| **GMP-4** | 3 | Yes |
| **GMP-1** | 3 | Yes |
| **MEP-3** | 4 | Yes |
| **MEP-4** | 4 | Yes |
| **MEP-1** | 4 | Yes |
| **Leu_Gmp-6** | 5 | Yes |
| **Leu_Gmp-7** | 5 | Yes |
| **Leu_Gmp-9** | 5 | Yes |
| **Leu_Gmp-11** | 5 | Yes |
| **Leu_Gmp-12** | 5 | Yes |
| **Leu_Gmp-13** | 5 | Yes |

Next, we tested our approach on Normal Progenitor and Leukemic Samples to find the haematopoietic stem cell (HSC), common myeloid progenitors (CMP), granulocyte macrophage progenitors (GMP), megakaryocyte erythroid progenitors (MEP), and leukaemic cells (L-GMP). We successfully organized the data set into five distinct classes with 100% accuracy, however, in (reference) author used HC to cluster this data set with rand index of 0.9740.

**Table S5: Clustering Results of CNS tumors**

| Field Description |  |
| --- | --- |
| Sample | CNS tumors data |
| MD MGlio Rhab Ncer PNET | Classification |
| Preprocessing and 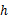 value | Normalize data with zscore and use 'cosine' distance at 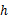 =7.4 |
| Rand Index | 0.9036 |
| Clusters Sequence  5= MD  1= MGlio  2= Rhab  3= Ncer  4= PNET | 5 5 5 5 5 5 5 **3** 5 5 1 1 1 1 1 1 1 **-1** 1 1 2 2 2 2 2 2 2 2 2 **-1** 3 3 3 3 **2** 2 4 4 5 **3** 4 4 |
| Note (the red color represents the misclassification and -1 is declared as noise) | |

In Tumor data set, the target was to find five classes with maximum accuracy.

**Table S6: Clustering Results of** Lung cancer data

| Field Description |  |
| --- | --- |
| Sample | Lung cancer |
| AD NL SQ COID | Identification of Cancer and Normal Tissue |
| Preprocessing and 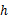 value | use 'minkowski' distance at 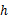 = 4.05 |
| Rand Index | 0.8845 Classes discovery, in case to separate normal lung from tumor, we achieved 98.4772% accuracy. |
| Clusters Sequence  1= AD  2= Normal Lung  3= SQ  4= COID | 1 1 1 1 1 1 1 1 1 1 1 1 1 1 1 1 1 1 1 1 1 1 1 1 1 1 1 1 1 1 1 1 1 1 1 1 1 1 1 1 1 1 1 1 1 1 1 1 **3** 1 1 1 1 1 1 1 1 1 1 1 1 1 1 1 1 1 1 1 1 1 1 1 1 1 1 1 1 1 1 1 1 1 1 1 1 **2** 1 1 1 1 1 1 1 1 1 1 1 1 1 1 1 1 1 1 1 1 1 1 1 1 1 1 1 1 1 1 **2** 1 1 1 1 1 1 1 1 1 1 1 1 1 1 1 1 1 1 1 **2** 1 1 2 2 2 2 2 2 2 2 2 2 2 2 2 2 2 2 2 3 3 3 3 3 3 3 3 3 1 1 1 1 1 1 1 1 1 3 1 3 4 4 4 4 4 4 4 4 4 4 4 4 4 4 4 4 4 4 1 4 |
| Note (the red color represents the misclassification) | |

**Table S7: Clustering Results of** mouse lung samples

| Field Description |  |
| --- | --- |
| Sample | mouse lung samples |
| MAL | Malignancy status (Normal/Tumor) |
| Rand Index | **1** |
| Preprocessing and 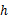 value | Normalize data with zscore and use 'minkowski' distance at 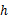=13 |

**Obtained Results**

|  | Sample | | MAL | Result | Correct? |
| --- | --- | --- | --- | --- | --- |
|  | N_MLUNG_1 | Normal | | Normal | Yes |
|  | N_MLUNG_2 | Normal | | Normal | Yes |
|  | N_MLUNG_3 | Normal | | Normal | Yes |
|  | N_MLUNG_4 | Normal | | Normal | Yes |
|  | N_MLUNG_5 | Normal | | Normal | Yes |
|  | T_MLUNG_1 | Tumor | | Tumor | Yes |
|  | T_MLUNG_2 | Tumor | | Tumor | Yes |
|  | T_MLUNG_3 | Tumor | | Tumor | Yes |
|  | T_MLUNG_4 | Tumor | | Tumor | Yes |
|  | T_MLUNG_5 | Tumor | | Tumor | Yes |
|  | T_MLUNG_6 | Tumor | | Tumor | Yes |
|  | T_MLUNG_7 | Tumor | | Tumor | Yes |

**Table S8: Clustering Results of Multi-a**

| Field Description |  |
| --- | --- |
| Sample | Multi-a |
| Br Pr Lu Co | Classification of sample into distinct classes |
| Preprocessing and 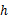 value | Normalize data with log2 and use 'Euclidian' distance at 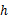 = 10 |
| Rand Index | 0.9714 |
| Clusters Sequence  1= Br  3= Pr  5= Lu  6= Co | 1 1 1 1 1 1 1 1 1 1 1 1 1 **6 5** 1 1 1 1 1 1 1 1 1 1 1 3 3 3 3 3 3 3 3 3 3 3 3 3 3 3 3 3 3 3 3 3 3 3 3 3 3 5 5 5 5 5 5 1 5 5 5 5 5 5 5 5 5 5 5 5 5 5 5 5 5 5 5 5 5 6 6 6 6 6 6 6 6 6 6 6 6 6 6 6 6 6 6 6 6 6 6 6 |
| Note (the red color represents the misclassification) | |

**Table S9: Clustering Results of Multi-b**

| Field Description |  |
| --- | --- |
| Sample | Multi-b |
| Br Pr Lu Co | Classification of sample into distinct classes |
| Preprocessing and 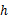 value | Normalize data with zscore and use 'Euclidian' distance at 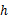 = 7.4 |
| Rand Index | 1 |
| Clusters Sequence  1= Br  2= Pr  3= Lu  4= Co | 1 1 1 1 1 2 2 2 2 2 2 2 2 2 3 3 3 3 3 3 3 4 4 4 4 4 4 4 4 4 4 4 |
|  | |

**Table S10: Clustering Results of Normal tissues**

| Field Description |  |
| --- | --- |
| Sample | Normal tissues |
| Breast Prostate Lung Colon Germinal Bladder Uterus Peripheral Kidney Pancreas Ovary Whole Cerebellum | Classification of sample into distinct classes |
| Preprocessing and 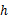 value | use 'Cosine' distance at 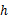 = 3.5 |
| Rand Index | 0.9443 |
| Clusters Sequence  1= Breast  2= Prostate  3= Lung  4= Colon  5= Germinal  7= Bladder  6= Uterus  8= Peripheral  9= Kidney  10= Pancreas  11= Ov**a**ry  12= Whole  13= Cerebellum | 1 1 1 1 -1 2 2 2 2 2 2 2 2 2 3 3 3 3 3 -1 -1 4 4 4 4 4 4 -1 4 4 4 4 5 5 -1 5 5 5 7 7 7 7 -1 -1 -1 6 7 7 7 6 7 8 8 8 8 8 9 9 9 9 9 9 9 9 9 9 9 9 10 10 10 10 10 10 10 10 10 -1 11 11 -1 11 12 12 7 12 12 13 13 13 |
| Note (the red color represents the misclassification and -1 is declared as noise) | |

**Table S11: Time complexity wise comparison of proposed method with famous clustering methods.**

| **Clustering Method** | **Time complexity** |
| --- | --- |
| CDP | 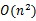 |
| Proposed Method | 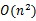 |
| Hierarchical Clustering | 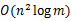 |
| K-means | 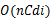,  (Where 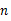is the number of d-dimensional vector, 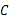 is the number of clusters, and 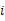 is the number of iteration until convergence ) |

**Table S12: RAND index for naïve-Bayes (NB) classifier, Hieratical clustering (HC), consensus clustering with hierarchical clustering (CC_HC_), consensus clustering with SOM(CC_SOM_), and proposed approach.**

| **Data Sets** | **BN** | **HC** | **CC_HH_** | **CC_SOM_** | **Proposed Method** |
| --- | --- | --- | --- | --- | --- |
| **Leukemia** | *1* | *0.648* | *1* | *0.721* | *1* |
| **Novartis multi-tissue** | *0.946* | *0.83* | *0.921* | *0.897* | *0.9899* |
| **St. Jude leukemia** | *0.971* | *0.949* | *0.948* | *0.825* | *0.9896* |
| **CNS tumors** | *0.632* | *0.628* | *0.549* | *0.429* | *0.9187* |
| **Lung cancer** | *0.904* | *0.307* | *0.310* | *0.233* | *0.8912* |
| **Normal tissue** | *0.655* | *0.572* | *0.572* | *0.487* | *0.7681* |

**References:**

1. Samaria, F. S. & Harter, A. C. Parameterisation of a stochastic model for human face identification. In *Applications of Computer Vision, 1994., Proceedings of the Second IEEE Workshop on*, 138–142 (IEEE, 1994)
